# Process evaluation of a cluster randomised intervention in Swedish primary care: using care managers in collaborative care to improve care quality for patients with depression

**DOI:** 10.1186/s12875-019-0998-4

**Published:** 2019-07-27

**Authors:** I. Svenningsson, E-L Petersson, C. Udo, J. Westman, C. Björkelund, L. Wallin

**Affiliations:** 1 Department of Primary Health Care, Institute of Medicine, The Sahlgrenska Academy, University of Gothenburg, Gothenburg, Sweden; 2 Region Västra Götaland, Närhälsan Research and Development Primary Health Care, Gothenburg, Sweden; 3 School of Education, Health and Social Studies, Dalarna University, Falun, Sweden; 4 Center for Clinical Research, Dalarna, Sweden; 5 Department of Health Care Sciences, Palliative Research Centre, Ersta Sköndal Bräcke University College, Stockholm, Sweden; 6 Division of Family Medicine and Primary Care, Department of Neurobiology, Care Sciences and Society, Karolinska Institutet, Huddinge, Sweden; 7 Department of Neurobiology, Care Sciences and Society, Division of Nursing, Karolinska Institutet, Stockholm, Sweden; 8 Department of Health and Care Sciences, The Sahlgrenska Academy, University of Gothenburg, Gothenburg, Sweden

**Keywords:** Accessibility, Care manager, Collaborative care, Continuity, Depression, Facilitator, Primary health care, Process evaluation

## Abstract

**Background:**

The collaborative care model with a care manager has previously generated beneficial results for patients with depression in terms of decreased burden of depression symptoms. A care manager function has been tested in Sweden in the PRIM-CARE RCT with successful results. The aim of the present study was to evaluate the process of implementing care managers in collaborative care for patients with depression in Swedish primary health care in the PRIM-CARE RCT.

**Methods:**

The study followed UK Medical Research Council guidance for process evaluation.

Field notes from the implementation of the PRIM - CARE RCT were used, as well as data collected from five focus group discussions with General Practitioners (*n* = 29) and three focus group discussions with care managers (*n* = 11). Data were analysed with content analysis.

**Results:**

Training sessions, careful preparation and extensive initial support to the care manager and staff at the Primary Care Centres were important ingredients in the implementation. The close access to facilitators, the recurrent peer support meetings, and the weekly newsletter strengthened the care manager function.

**Conclusions:**

A complex intervention adapted to the Swedish primary care context focusing on a care manager function for patients with depression could be performed through a stepwise implementation process. Financial support from the health care regions included in the study helped to reduce the impact of identified barriers. This process evaluation has revealed new and important knowledge for primary care development concerning infrastructure and organization building, knowledge sharing, and facilitating factors and barriers.

**Trial registration:**

NCT02378272 Care Manager – Coordinating Care for Person Centered Management of Depression in Primary Care (PRIM – CARE). Registered March 4 2015. Retrospectively registered.

**Electronic supplementary material:**

The online version of this article (10.1186/s12875-019-0998-4) contains supplementary material, which is available to authorized users.

## Background

The Collaborative Care Model (CCM**)** based on Wagener’s Chronic Care Model [1] is a pragmatic and cost-effective way of working with people with mental health problems in primary health care (PHC), where collaboration between professions in the team is an important component [2]. A central role within the CCM is held by the care manager, who interacts with the patient and facilitates the care process through engaging the patient by self-management support, coordinating the providers of patient care, monitoring patient progress, and assessing the patient’s adherence to pharmaceutical and psychological treatment [2–5]. The CCM model has generated beneficial results for patients with chronic diseases and for patients with depression in terms of decreased burden of depression symptoms [6, 7]. Depression has a large impact on public health worldwide, and more than 300 million people are affected, resulting in 50 million years lived with disability [8]. In Sweden, one third of the population will be affected during the lifetime, and the prevalence of comorbid depression and somatic illness is common [9]. More than 70% of people with depression are treated in primary health care [10]. Depression causes loss of quality of life and decreased functional ability, with economic and social consequences and increased risk of suicide [11]. Therefore, these patients need to be taken care of at an early stage [12]. As PHC is the first instance where people with depression seek care, there is a need to develop methods to increase accessibility and continuity in care [9].

In a systematic review by the Swedish Agency for Health Technology Assessment and Assessment of Social Services, it was concluded that the care manager function had generated beneficial results in previous studies, and there was a need to evaluate how a care manager organization would work in the Swedish PHC setting [13]. Our team set out to do so, and in a cluster randomised controlled trial, the PRIM-CARE RCT, the effect of supplementing the ordinary primary care team with a care manager was evaluated [3]. Out of 210 primary care centres (PCCs) in two Swedish health care regions, 23 PCCs participated in the trial, with 11 as intervention sites and 12 as control sites. Registered nurses were specifically trained for taking the role of a care manager, focusing on accessibility and continuity in care for patients with depression. The care manager was a practice-based staff member with direct patient contact. The care manager’s responsibility was to create a care plan together with the patient and to support and continue contact via an initial face to face meeting, followed by 6–8 telephone contacts during a 12 week period. In addition, the patient could contact the care manager in between the scheduled telephone calls if needed. Every contact should consist of person-centered communication regarding the patient’s current depression symptoms, evaluated with a self-assessment instrument (MADRS-S) [14, 15], as well as behavioural activation. The care manager was to work in close cooperation with the general practitioner (GP) and other professionals in the team during the depression episode [2]. Care managers were to coordinate the activities described above and participate in both the clinical and nonclinical aspects of care [16]. The patients in the control arm received care as usual (CAU), i.e. cognitive behavioural therapy, interpersonal therapy, and/or antidepressants in accordance with The Swedish National Guidelines for Depression and Anxiety Disorders [9].

Findings of the PRIM-CARE RCT, reported in detail by Björkelund et al. 2018, showed positive results regarding reduction of depression symptoms, improved quality of life, and significantly increased rate of returning to work for those patients who had been provided with a care manager [3]. The care manager intervention was also shown to be highly cost-effective [17].

Parallel to the trial, we performed a process evaluation. The Medical Research Council (MRC) in UK emphasises that it is vital to understand how interventions work in practice and how they produce change. MRC’s guidance for process evaluation focuses on the relationships between causal assumptions regarding the effect of the intervention, its implementation, the context, mechanisms of impact, and outcomes [18]. Thus, to enhance the opportunities to understand and explain the positive outcomes of the PRIM-CARE RCT, this article will report on the implementation process of the collaborative care organization in Swedish primary health care.

## Aim

The aim of this study was to evaluate the process of implementing care managers in collaborative care for patients with depression in Swedish primary health care.

## Methods

### Study design

We applied the guidance for process evaluation suggested by Moore et al. [18] focusing on the context for the implementation and how the implementation was accomplished, as well as on the mechanisms of impact for the achieved results [18], during the time frame of 14 months. An explorative design with qualitative methods was used.

### Setting

The PRIM-CARE RCT was conducted in two Swedish healthcare regions with both urban and rural areas. There were 11 intervention PCCs with 4000 to 18000 people listed per PCC, staffed by various professions, primarily registered nurses and GPs. To be included, the PCCs were required to have at least two permanently employed GPs.

### Participants

Care managers: Eleven registered nurses, one at each intervention PCC, working 20–25% of full-time as care managers.

GPs: A total of 29 working at the intervention PCCs.

### The implementation programme

The plan for the implementation programme consisted of several components, described as follows.

#### Care manager tour

Initially, the research team visited every intervention PCC to inform the PCC manager, staff and the assigned care manager about the study and the care manager function and to discuss any issue. Every PCC had its own financial budget for which the PCC manager was responsible and had the full responsibility to plan and coordinate the resources. Each PCC had in advance selected which nurse to be assigned to the care manager role.

#### Education sessions

The GPs, employed by the PCCs, were invited to a one-day session as part of their duties and the care managers were invited to a three-day training session before the start of the intervention, followed by two one-day sessions during the initial part of the intervention period. The care manager training comprised: 1) general education regarding mental health treatment in the primary care context according to the Swedish National Guidelines on depression and anxiety syndromes [9] and 2) the components of the care manager function [19].

#### Peer support meetings

Peer support meetings were offered to all care managers every second month. These support meetings provided opportunities to meet and discuss their experiences of care management and for jointly developing the care manager function.

#### Newsletter

Every second week a newsletter was e-mailed to all care managers and PCC managers with information about the development of the care manager project and how many patients every PCC had included.

#### Workshops at the PCCs

In addition, a web-based workshop was created for initiating a discussion among staff at each PCC focusing on how the patients with depression were taken care of at the PCC.

#### Facilitators

Four specially trained research nurses acted as facilitators, providing continuing support for the care managers. The support, for example, included regularly visiting each care manager and being available by telephone and e-mail daytime.

The research team, consisting of people with different professional backgrounds, was prepared to provide support to the facilitators and the PCCs during the entire intervention period.

#### Financial reimbursement

The health care regions provided financial reimbursement for the intervention PCCs covering the care managers’ (part-time) salary.

### Data collection and data analysis

This process evaluation comprised a considerable amount of data, see Table 1. Data collected from the focus group discussions were analysed using qualitative content analysis [20], as were the field-notes, documents, and the summarised notes from meetings and workshops. Initially, all transcriptions were read several times by three of the authors (IS, ELP and CU) to get a sense of the meaning as a whole. The first impressions were then discussed among the authors, before the analysis process continued by coding the transcriptions, i.e., labelling units of texts, guided by the aim of the study. Codes with similar meaning were grouped into preliminary subcategories before further aggregated into categories. The analysis process was primarily led by three of the authors (IS, ELP and CU); ongoing discussions among all the authors were held during the entire analysis process to achieve a transparent and trustworthy analysis.

**Table 1:** Data sets used, related to each component of the process evaluation framework

Key component	Description	Data sources
Context	Contextual factors of importance for if and how the intervention was implemented	3 focus groups with care managers during the intervention (*n* = 8) 5 focus groups with GPs during and after the intervention (*n* = 29) Field notes from research team/facilitators Notes from meetings (e.g. with PCC managers) Field notes and documents from the care manager training Observations from care manager tour (care manager and colleagues/manager at the PHCC)
Implementation	What the intervention consisted of and how it was delivered	Weekly newsletters Notes from peer support meetings Field notes during the intervention period Reports from PCCs
Mechanisms of impact	Study group responses to and interactions with the intervention	3 Focus groups with care managers (n = 8) 5 Focus groups with GPs (n = 29) Notes from peer support meetings Field notes from meetings with facilitators Notes from research group meetings Documents from the training of care managers Field notes from care manager tour

## Results

The results presented below are organized according to the MRC framework [18].

### Contextual factors

In Sweden, GPs traditionally have an influential position at the PCCs. According to the focus group discussions with the care managers, this was also the case at participating PCCs. They emphasised the need for the GPs to be actively involved and engaged for the care manager to succeed. There was a shortage of GPs working at the PCCs, which reflects the general situation in Swedish primary health care. As a result, the types of employment varied among the GPs and temporary employments were common, causing a lack of collaboration with the care manager.

There was also a variation in the PCCs’ practice culture, for example how they identified and handled patients with various mental health problems. Some PCCs could provide a psychosocial team, including a psychologist, a social worker, and a psychiatric nurse, while other PCCs had a shortage of competences working with mental health problems.

### Implementation issues

Overall, the procedure for supporting the implementation of the care manager was followed as planned, using the care manager tour, peer support meetings, facilitators, etc. During the care manager tour, information was collected about each PCC’s prerequisites, which was used to tailor the support when implementing the care manager function.

All PCCs participated in the education programme with the selected care manager and one of the GPs at each PCC. All PCCs also remained in the study throughout the entire intervention period, except one where the care manager unexpectedly terminated the employment at the PCC without being replaced. At another PCC, the care manager went on sick leave for two months, but was replaced by another nurse with good knowledge of the care manager function.

Attendance at the peer support meetings was prioritised by the care managers. All of them were actively participating, sharing their experiences and discussing the problem areas that had emerged. The facilitators maintained their weekly contact with the care managers as planned. Some of the PCCs needed extra support regarding the care manager function, which led to extra visits by the facilitators and the research team, see Table 2. In a meeting with PCC managers, the discussion indicated an ambiguity regarding the care manager function. Therefore, it was necessary during the meeting to clarify the function, focusing on the care manager function in the collaborative care team. The regular contact with the facilitators and support from the research team ensured that the intervention plan, i.e. number and content of contacts with the patient, was followed accordingly.

**Table 2:** Issues experienced by care managers and how they were handled

Problems identified by the care managers in the initial phase	How the problems were handled by the facilitators
Feeling insecure in the care manager role.	Frequent proactive telephone contacts and visits when needed.
	Easy access to facilitators in between scheduled meetings.
Feeling insecure regarding the care manager method and what was expected of them as care managers.	More proactive telephone contacts and visits when needed.
	Repeated information and support regarding the method, e.g. how to conduct follow-up telephone contacts with the patients and how to use the self-assessment scale.
Feeling insecure about meeting the first patient.	Supporting the care manager by preparing her/him before the first patient meeting by discussing any uncertainties and questions.
	Facilitators meeting the first patient together with the care manager.

Although the implementation programme was carefully planned, there was a need for continuous development, and different adaptions were made along the way. These adjustments, adapted to clinical reality, were carefully discussed by the research team before implemented. For example, the care managers described that they could not always reach the patients at their scheduled telephone contacts. To address this issue, it was decided that the care manager should initially ask the patient, “What do I do if you don’t answer the phone?” Additionally, an information sheet was prepared with information to the patient, where the care manager function was briefly described: how to contact the care manager when needed, what to expect of the care manager contact, and agreed upon dates for telephone contacts (See Additional file 1). Further, a standardised care manager template for documentation of the care plan in the electronic patient record was produced.

### Mechanisms of impact

In the analysis of the data, facilitating factors and barriers were identified, see Table 3.

**Table 3:** Facilitating factors and barriers

Facilitating factors	Barriers
Preparation and initial support	Lack of support
Educational package	GP as gatekeeper
Access to facilitators	Unclear care manager function
Care manager peer support	
Newsletter as continuous information	
Economic remuneration	

### Facilitating factors

#### Preparation and initial support

The care manager tour paved the way for the implementation of the care manager function. PCC staff perceived that the information was provided and discussed in a pragmatic way, in a dialogue where all staff at the PCC had an opportunity to have a say about the function and its use in patient care.

#### The training package

The care managers expressed that the training provided new knowledge about depression, dialogues, suicide, medication etc., which was perceived as valuable for themselves and when meeting the patients. The care managers appreciated being able to influence the content of the lectures. In addition, since the training programme was performed over time, the care managers were able to discuss complex situations arising during the course of their work as care managers.

*Everything in the education was very useful, especially the lectures. I’ve received so much support from you and you were so committed to help. (Focus Group (FG) 1 care manager).*

#### Access to facilitator

The care managers appreciated when the facilitators visited them in their clinical practice at the PCC and joined in their first patient meeting. This was especially important when the care manager felt insecure in the function and hesitant to meet the first patient. Although there were scheduled meetings between the facilitators and care managers, the opportunity to contact the facilitator also in between meetings was described as crucial by the care managers, especially because the care manager function was new to all at the PCCs.

*I received great support from the facilitator, who was also together with me when meeting the first patient. The easy access was so important, you know, to just be able to call or e-mail her/him (FG 2 care manager).*

#### Care manager peer support

Since care managers experienced that they were alone in their function at the PCC, peer support from other care managers was important. Sharing experiences in how to act as care manager and clarifying and defining the function in the collaborative care team became essential at the peer support during the course of the project.

#### Continuous information through newsletter

The newsletter every second week with updates about how the intervention progressed served as a motivation for the care manager. The GPs also found the continuous information important, and it inspired further involvement.

*The newsletter made it possible for us to follow the project over time so we were all informed and involved in the progress. (FG 1 GP).*

#### Financial remuneration

The healthcare regions provided financial support for the implementation of the care manager function. Support covered the salary costs of the new care manager function, enabled participation, and made it possible to replace the ordinary part-time nurse engagement that the care manager nurse allocated to patients with depression.

### Barriers

#### Lack of support

When the care manager experienced lack of support from the PCC manager, it was difficult to get the function to work properly since the care manager had to struggle her/himself to make other practitioners understand the function. At some PCCs the manager experienced difficulties in engaging and involving the GPs, which influenced the implementation negatively.

*… and then we have this enormously strong group of doctors. Many of them have worked together for 20 years. Then it’s really hard to tell them to change the way they do their job. It’s obvious my boss (the PCC manager) is fighting elephants (FG 2 care manager).*

#### The GP as gatekeeper

Although the GPs appreciated the collaboration and support in the work with patients with depression, the GPs were used to having responsibility for this group of patients, which sometimes led to them acting as gatekeepers, i.e. they kept the patients to themselves. The newly educated and younger GPs were perceived by the care managers as more open to collaboration than the more experienced GPs. The GPs underlined that habits and routines took time to change. In addition, if the care manager was not located in the same building as the GPs, they often forgot that they had a care manager in the team.

#### Unclear care manager function

The care managers expressed that they sometimes experienced the description of the function as vague and asked for a clearer definition. The care managers did not describe having to put other responsibilities aside in order to work as a care manager, although they expressed it was sometimes difficult to separate the function from other professionals’ responsibilities in the collaborative care team. Sometimes other team members who did not know about the patient inclusion criteria for the study wanted the care managers to also meet other patients, e.g. patients with long-term and more chronic depression symptoms and patients with anxiety disorders. The care managers sometimes asked for more information from the research team that could be handed over to the team members in order to give them a better understanding of what the care managers were assigned to do.

*You know, it’s important to have something in writing that clearly says what we actually do as a care manager (FG 1 care manager).*

## Discussion

This process evaluation addressed the implementation of a complex collaborative care intervention consisting of a care manager for patients with depressive disorders in the Swedish primary health care. The parallel RCT showed convincing results regarding patient recovery, return to work [3], and cost effectiveness for the society [17]. The findings indicate that careful preparation and extensive initial support to the care manager and staff at the PCCs were important ingredients for successful implementation. The close access to facilitators, the recurrent peer support meetings, and weekly newsletter seem to have strengthened the care manager’s role.

Both the care managers and GPs expressed great interest in the education programme and were eager to participate. A majority followed the programme throughout the project. The care managers also emphasised the need for continued support and information as the project proceeded and new questions arose, e.g. enhanced clarification about the care manager role. The care manager focus group discussions indicated that the PCC manager had an important role in enabling and supporting the care manager. Strong leadership support has in a similar way been shown to impact positively when implementing collaborative care [21]. The facilitators worked intensively to support the process, e.g. through continuing contacts and availability for questions at any time during the day. This support was considered necessary to strengthen the care manager role at the PCC. Other studies have also shown the importance of facilitators when implementing new programmes [22] and when sustaining care managers in regular care [16, 23]. A more extensive facilitation support, also including the PCC managers, might have increased the positive effects of implementing the care manager function.

The findings indicate that the broad preparation and flexibility during the care manager introduction at the PCCs was essential. This is in line with Overbeck et al. [24] findings, showing the need to adapt and tailor the model of collaborative care to the resources and the target group at the specific setting. Also similar to Overbeck et al. [24], peer support, i.e. meeting others in the same situation and sharing each other’s experiences, was found to be fundamental for the development of the care manager role. The implementation of the care manager required flexibility and new tools had to be developed. One of the new tools was the information sheet, which was beneficial for providing added safety for the patient. Another new tool was a care manager template in the electronic patient record, which secured the documentation and also made the care plan available to the collaborating team. The newsletter appeared to motivate the care managers and prepared them for the collaborative work. It also provided an opportunity for them to gain a picture in common of the care manager function.

Adequate resources are one main feature of the context when implementing innovative ways of working [25]. In our study the PCC managers underlined the benefit of the targeted financial resources from the region for overcoming obstacles, which gave opportunities to replace the nurse who took on the role of care manager. Previous studies have shown that economic incentives are important when implementing collaborative care [21, 23, 26] and for encouraging change and improving long term results [27].

According to Ashcroft [28], the culture in the PCC has an important influence on how mental health care is delivered. The management of patients with mental health disorders differed among the PCCs in our study. Meeting notes showed that some PCCs had difficulties in identifying this group of patients with the risk of not offering them the care manager services. The different ways of handling these patients at the PCCs might have constituted an obstacle when implementing the care manager function.

The GPs’ involvement in the collaborative work at the PCCs varied but was found vital. Coupe et al. [29] have reported that GPs can constitute a serious barrier if they do not realise their crucial role in collaborative care. A way to improve the relationship between the care manager and the GP could be to tailor the collaboration to meet both care managers’ and GPs’ particular preferences [30]. Another problem revealed by our study was that the shortage of permanently employed GPs hindered continuity in collaboration between care managers and GPs. The constant influx of new GPs also complicated the care managers’ work due to their lack of awareness about the care manager function. This situation caused a need for repeated information about the care manager function to any new GP.

Despite the efforts to clearly describe and educate about the care manager role, it was sometimes difficult for the care manager to fully grasp what the role entailed. This also influenced the collaboration at the PCC. Svenningsson et al. [31] found that the care managers were disappointed when the GPs did not believe in the usefulness of the care manager. Winklerfeldt et al. [32] showed in a qualitative study that some of the GPs participating in the RCT perceived themselves as a sort of care manager and therefore were not offering this service. This indicates the importance of informing GPs about the care manager function and the benefits with a care manager in the collaborative care team. We believe that this extended collaborative care team could lead to an increased accessibility and continuity for the patient, as well as high-quality self-management support.

### Strengths and limitations

This process evaluation was planned together with the RCT. We included data from a careful qualitative analysis as well as detailed documentation from the implementation, which gave a broad picture of the implementation process. Credibility and reliability of the coding was strengthened by the fact that three of the authors (IS, ELP, CU) took part in the reflexive and critical discussions that occurred when moving back and forth between preliminary codes and original descriptions. Codes were kept close to the original descriptions, which enhanced transparency and facilitated agreement on the final codes among all authors. However, this process evaluation has some limitations. The authors were engaged in the design, planning, and accomplishment of the implementation of the Care Manager organization, which might have biased the analysis. However, by following the MRC guidance [18] on performing process evaluation, this potential bias has been reduced. Another limitation is that notes and diaries could have been more extensive and the facilitator contacts more strictly documented.

## Conclusions

A complex intervention adapted to the Swedish primary care context focusing on a care manager function for patients with depression could be performed through a stepwise implementation process. Overall, the care manager function was relatively smoothly adapted to suit Swedish PCCs. Strengths in the implementation process, such as careful preparation and an extensive training programme, peer support, continuous information, and financial support appear to have been able to reduce the impact of identified barriers. In this process, information from the care managers, GPs and facilitators was important for continuously adjusting the implementation to be more effective. This process evaluation has revealed new and important knowledge for primary care development concerning infrastructure and organization building, knowledge sharing, facilitating factors and barriers.

## Additional file

Additional file 1: Information to the patient. (DOCX 45 kb)

## Data Availability

The datasets used and/or analysed during the study are available from the corresponding author on reasonable request.

## Abbreviations

CCM: Collaborative Care Model
GP: General practitioner
MRC: Medical Research Council
PCC: Primary care centre
PHC: Primary Health Care
