# Supplementary material for: Process evaluation of a cluster randomised intervention in Swedish primary care: using care managers in collaborative care to improve care quality for patients with depression
Source: BMC Fam Pract. 2019 Jul 27;20:108. doi: 10.1186/s12875-019-0998-4 (PMC6660943; doi:10.1186/s12875-019-0998-4)
Supplement: Supplementary file 1 — Information to the patient. (DOCX 45 kb) [file 12875_2019_998_MOESM1_ESM.docx]

| Your Care Manager is:  You can reach your Care Manager at this phone number:  First appointment:  ___________  Phone appointments:  1 ___________  2 ___________  3 ___________  4 ___________  5 ___________  Final appointment:  ________________ |  | Care Manager  in  Primary Health Care Support for depression |
| --- | --- | --- |
| **Information about the Care Manager function** The Care Manager gives support and is the contact person for patients with depression at the Primary Care Centre.  The Care Manager cooperates with the GP and other professionals.  The Care Manager follows symptoms, treatment, and provides advice on self-care.  The Care Manager is part of the Primary Care Centre's mental health management. |  | What does the Care Manager do for you?  - Offers regular contacts - Follow-up of your treatment - Follow-up regarding your wellbeing - Provides advice and support - Ensures that you get help when needed |
